# Predictive biomarkers and initial analysis of maternal immune alterations in postpartum preeclampsia reveal an immune-driven pathology

**DOI:** 10.3389/fimmu.2024.1380629

**Published:** 2024-04-30

**Authors:** Camille Couture, Marie-Eve Brien, Jade Rechtzigel, SuYun Ling, Cecilia Ledezma-Soto, Gilberto Duran Bishop, Ines Boufaied, Dorothée Dal Soglio, Evelyne Rey, Serge McGraw, Charles H. Graham, Sylvie Girard

**Affiliations:** ^1^ Department of Obstetrics and Gynecology; Department of Immunology, Mayo Clinic, Rochester, MN, United States; ^2^ Department of Microbiology, Infectiology and Immunology, Université de Montréal, Montreal, QC, Canada; ^3^ Sainte-Justine Hospital Research Center, Montreal, QC, Canada; ^4^ Department of Biochemistry, Université de Montréal, Montreal, QC, Canada; ^5^ Department of Pathology and Cellular Biology, Université de Montréal, Montreal, QC, Canada; ^6^ Department of Obstetrics and Gynecology, Université de Montréal, Montreal, QC, Canada; ^7^ Department of Biomedical and Molecular Sciences, Queen’s University, Kingston, ON, Canada

**Keywords:** postpartum preeclampsia, immunology, inflammation, exhaustion, maternal circulation, single-cell RNA sequencing, placenta, biomarkers

## Abstract

**Introduction:**

Postpartum preeclampsia (PPPE) is an under-diagnosed condition, developing within 48 hours to 6 weeks following an uncomplicated pregnancy. The etiology of PPPE is still unknown, leaving patients vulnerable and making the identification and treatment of patients requiring postpartum care an unmet need. We aimed to understand the immune contribution to PPPE at the time of diagnosis, as well as uncover the predictive potential of perinatal biomarkers for the early postnatal identification of high-risk patients.

**Methods:**

Placentas were collected at delivery from uncomplicated pregnancies (CTL) and PPPE patients for immunohistochemistry analysis. In this initial study, blood samples in PPPE patients were collected at the time of PPPE diagnosis (48h-25 days postpartum; mean 7.4 days) and compared to CTL blood samples taken 24h after delivery. Single-cell transcriptomics, flow cytometry, intracellular cytokine staining, and the circulating levels of inflammatory mediators were evaluated in the blood.

**Results:**

Placental CD163+ cells and 1^st^ trimester blood pressures can be valuable non-invasive and predictive biomarkers of PPPE with strong clinical application prospects. Furthermore, changes in immune cell populations, as well as cytokine production by CD14+, CD4+, and CD8+ cells, suggested a dampened response with an exhausted phenotype including decreased IL1β, IL12, and IFNγ as well as elevated IL10.

**Discussion:**

Understanding maternal immune changes at the time of diagnosis and prenatally within the placenta in our sizable cohort will serve as groundwork for pre-clinical and clinical research, as well as guiding clinical practice for example in the development of immune-targeted therapies, and early postnatal identification of patients who would benefit from more thorough follow-ups and risk education in the weeks following an uncomplicated pregnancy.

## 1 Introduction

Preeclampsia (PE) is an important cause of maternal morbidities and mortality, with its predominant presentation in the antepartum/intrapartum period being widely studied (1). However, the postpartum manifestation of PE, also associated with major maternal morbidities, is poorly understood and patients are largely underinformed about its potential development following pregnancy. Postpartum preeclampsia (PPPE) is defined as a new onset of PE, characterized by *de novo* hypertension and end-organ damage, but occurring between 48 hours to 6 weeks after a seemingly uncomplicated pregnancy (2–4). It has proven challenging to determine the precise incidence of PPPE, as it is commonly under-diagnosed, and there is a lack of consistent follow-up in the postpartum period of normotensive pregnancies; however, studies have estimated the incidence to be between 3-27% for hypertensive disorders, including PPPE, in the postpartum period (5–7). Importantly, there is currently no way to identify patients before symptom onset and patients who develop PPPE are at risk for severe morbidities both in the short and long term for example having seizures, stroke, and pulmonary edema as well as having a higher risk of cardiovascular diseases later in life (8–11).

A unique feature distinguishing PPPE from PE is the placenta, which in PE has been shown to be an essential driver of the pathology since the end of pregnancy with its removal is the only known curative treatment (12). In the case of PPPE, the placenta cannot explain the postpartum occurrence of the disease, suggesting that a different, and still unknown, pathophysiologic mechanism is involved. A widely accepted feature in PE is an activated and altered maternal immune system, which has recently been demonstrated to be only partly shared with PPPE and provides an avenue that needs further characterization (13, 14). Although some features are shared, work by us and others has demonstrated that the maternal immune profiles of patients with PPPE are generally distinct from those with PE, indicating that the postpartum incidence is a distinct pathology (7, 13, 14).

In this study, we aimed to investigate the placenta at delivery and blood pressure measurements in the 1^st^ trimester to uncover potential non-invasive biomarkers while assessing their predictive capabilities for the early identification of women at risk of developing the condition. Furthermore, we investigated the circulating maternal immune cells and inflammatory mediators in PPPE at the time of diagnosis (namely 48h-25 days postpartum), to obtain an in-depth understanding of the immune changes observed. Due to the restricted follow-ups in the post-partum period for uncomplicated pregnancies, acquiring time-matched samples beyond 24 hours is challenging. We have addressed this in our analysis through correlations between each mediator/immune cell and PPPE onset.

## 2 Method of study

### 2.1 Patient and sample collection

Patients (n=96) were recruited at the CHUSJ either at the time of delivery with uncomplicated pregnancies (N=54, CTL) or when admitted for PPPE (N=42) and gave written informed consent. PPPE was defined as *de novo* hypertension (140/90mmHg) with end-organ damage, most commonly proteinuria (≥300mg/day), in the postpartum period (48h to 25 days after birth following an uncomplicated pregnancy) (13, 15). Demographic and obstetrical data were obtained through clinical chart review. Maternal blood samples were collected 24 hours after delivery (CTL) or at the time of diagnosis (PPPE). Approximately 8 ml blood was collected in 10mL K2 EDTA tubes (ThermoFisher) and processed immediately. From whole blood, 150µl was transferred to a round bottom tube for immunolabelling (see below) and the remaining blood was centrifuged at 845g (10 minutes, 4°C) and plasma was collected, aliquoted, and stored at −80°C. The residual blood was used for peripheral blood mononuclear cells (PBMC) isolation (see below).

### 2.2 Peripheral blood mononuclear cell isolation

Residual blood was diluted with PBS-10%FBS in a 1:1 ratio and added to Sepmate-50 tubes (Stemcell) containing 15mL Lymphoprep density gradient medium (StemCell). Tubes were centrifuged at 1200g for 10 minutes. PBMCs were collected and diluted with PBS for a final volume of 45mL and centrifuged at 300g for 10 minutes. Supernantants were removed and the pellet was resuspended with 10mL of PBS. A 10µl aliquot was taken and combined with 10µl trypan blue solution (Bio-Rad) for cell count on a Bright-Line hemacytometer (Hausser Scientific). The resuspended pellet was centrifuged at 400g for 10 minutes and the supernatant was discarded. Pellets were resuspended with FBS-10%DMSO at a final concentration of 2M cells/mL and aliquoted to be frozen down slowly at -80°C for 24-48 hours and then transferred to liquid nitrogen for long term storage.

### 2.3 Immune cell analysis by flow cytometry

Whole blood (150μl) was immunolabeled for 15 minutes at room temperature using the following antibodies [with catalog numbers; concentration]: 5μl for each of the following APC-Cy7-CD4 [557871; 0.2mg/ml], PerCP-Cy5.5-CD8 [560662; 0.05mg/ml], Alexa Fluor 700-CD14 [557923; 0.2mg/ml], BV605-CD25 [562660; 0.025mg/ml], PE-Cy7-CD196 [560620; 0.2mg/ml], BV421-CD194 [562579; 0.2mg/ml], PE-CF594-CD183 [562451; 0.2mg/ml], PE-CD127 [557938; 0.2mg/ml], V500-CD19 [561121; 0.2mg/ml] and 20μl of the following: FITC-CD3 [555339; 0.05mg/ml], and APC-CD56 [555518; 0.025mg/ml] (all from BD Biosciences) and 35μl buffer solution (BD Canada). After the immunolabelling was completed, red blood cells were lysed (0.06M NH4Cl, 4mM KHCO3, 0.052mM EDTA), and peripheral blood mononuclear cells were washed with PBS, PFA-fixed, and analyzed using a flow cytometer (LSR Fortessa, BD Biosciences). A minimum of 300,000 events were acquired per sample. Data analysis was performed using FlowJo (Tree Star Inc).

### 2.4 Intracellular cytokine staining of PBMCs

Intracellular cytokine staining was performed on a subset of our patients to assess immune cell mediator production. PBMCs were thawed and resuspended in RPMI/10% FBS (Wisent). Cells were plated in 96-well plates for a final count of 1 million/well, and treated for 4h (37°C, 5% CO_2_) with one of the following: untreated (PBS/1%FBS), lipopolysaccharide (LPS from *E. coli*, 100ng/ml), monosodium urate crystals (MSU, 100mg/ml) or phorbol myristate acetate (PMA, 50ng/ml). After 1h of incubation, GolgiPlug (BD Biosciences) was added to wells. Cells were washed and incubated with the extracellular antibody cocktail [with catalog numbers; concentration] including 5μl of each of the following: (anti-CD3 [564001; 0.1mg/ml], anti-CD4 [557871; 0.2mg/ml], anti-CD8 [560662; 0.05mg/ml], anti-CD14 [557923; 0.2mg/ml], and anti-CD16 [563690; 0.2mg/ml] (all from BD Biosciences), viability dye (ThermoFisher) and brilliant stain buffer (BD Biosciences) for 20 minutes at room temperature. Cells were washed and were fixed and permeabilized in 200μl Cytofix/Cytoperm solution (BD Biosciences) for 20 minutes at room temperature, followed by a washing step with Perm/Wash (1:10 dilution; BD Biosciences). Cells are then incubated with an intracellular antibody cocktail [with catalog numbers] including 5μl of each of the following (anti-IL-10-PeCy7 [501420; 0.02mg/ml], anti-IFNγ-Pe [506507; 0.05mg/ml], anti-IL-1β-FITC [508206; 0.05mg/ml] (Bio legend), and anti-TNFα-BV421 [562783; 0.2mg/ml], anti-IL-12-APC [554576; 0.2mg/ml] (BD Biosciences), with brilliant stain buffer (BD Biosciences) for 20 minutes at room temperature. Cells were washed, resuspended in PBS/1%FBS, and passed by Flow Cytometry (LSR Fortessa, BD Biosciences) 300,000 events were acquired per sample. Gating was performed on live cells and data analysis was performed using FlowJo (Tree Star Inc).

### 2.5 Detection of inflammatory mediators in the maternal plasma

Plasma was analyzed for a panel of immune mediators via multiplex analysis (*Human Cytokine 48-Plex Discovery Assay*, Eve Technologies). This panel included the following mediators: sCD40L, EGF, Eotaxin, FGF2, Flt3 ligand, Fractalkine, GCSF, GMCSF, GROA, IFNA2, IFNγ, IL1α, IL1β, IL1RA, IL2, IL3, IL4, IL5, IL6, IL7, IL8, IL9, IL10, IL12p40, IL12p70, IL13, IL15, IL17A, IL17E, IL17F, IL18, IL22, IL27, IP10, MCP1, MCP3, MCSF, MDC, MIG, MIP1α, MIP1β, PDGFAA, PDGFAB/BB, RANTES, TGFα, TNFα, TNFβ, VEGFA.

### 2.6 Single-cell RNA sequencing

PBMCs were sequenced using 10X Chromium Next GEM Single Cell 3’ Reagent Kits v3.1 (dual index) with feature barcode technology for cell multiplexing at the McGill Genome Center using the Illumina NovaSeq6000 S4 v1.5, PE100 (16). The Chromium protocol was followed, including GEM generation and barcoding, followed by GEM-RT cleanup and cDNA amplification, gene expression library construction, and lastly, cell multiplexing library construction and sequencing. Libraries were prepared to obtain 5000 cells/patient and to sequence approximately 30,000 reads/cell. Files were demultiplexed using CellRanger (17) pipeline version 7.0.1 default parameters, whereby only cell-associated barcodes assigned exactly one cellular multiplexing oligo (CMO) were assigned to a sample and with reference to the *Homo sapiens* genome, build GRCh38 2020-A.

### 2.7 Single cell RNAseq analysis

Data were analyzed with the ROSALIND® platform (https://rosalind.bio/), with HyperScale architecture. Quality scores were assessed using FastQC (18). Cell Ranger was again used to count UMIs, call cell barcodes, and perform default clustering. Individual sample reads were normalized via Relative Log Expression (RLE) using DESeq2 R library (19). Read Distribution percentages, violin plots, identity heatmaps, and sample MDS plots were generated as part of the QC step using RSeQC (20). DEseq2 was also used to calculate fold changes and p-values and perform optional covariate correction. The clustering of genes for the heatmap of differentially expressed genes was done using the PAM (Partitioning Around Medoids) method using the fpc R library (21). Final clustering was done using Seurat graph-based method. Hypergeometric distribution was used to analyze the enrichment of pathways, gene ontology, domain structure, and other ontologies. The topGO R library (22) was used to determine local similarities and dependencies between GO terms to perform Elim pruning correction. Enrichment was calculated relative to a set of background genes relevant to the experiment. BioPlanet and biological processes from the GO terms were the only pathway catalogs used for our analyses.

### 2.8 Histological analysis of the placenta

Placental biopsies, at least four, were obtained and included the entire thickness of the placenta. For immunohistochemistry, sections were processed as previously described (23). Briefly, sections were deparaffined and incubated with primary antibody overnight at 4°C. The following antibodies were used (dilution; concentration; catalog number, company): mouse anti-CD163 (1:100; 0.2mg/100μl; MCA1853, Bio-Rad), mouse anti-HO2 (1:50; 0.74mg/mL; NBP2-01407, Novus Biologicals), and rabbit anti-LC3b (1:100; 1mg/mL; NBP2-46892, Novus Biologicals). Sections were then washed with PBS, exposed to the matched horseradish peroxidase (HRP)-conjugated secondary antibody (goat anti-mouse IgG-HRP (1:100; 1mg/mL; 1706516, Bio-Rad), or goat anti-rabbit IgG-HRP (1:100; 1mg/mL; 1706515, BioRad), for 45 minutes at room temperature, and washed before the signal was revealed using DAB (Sigma) and counterstained with hematoxylin. Slides were imaged using a microscope (Revolve, Discover Echo), where 6–8 pictures were randomly taken within the placental villi or captured using a slide scanner (Axioscan, ZEISS). Image analysis was performed using ImageJ (NIH Image). For immune cells, counting was performed by a blinded individual, and data were reported as the number of positive cells by 1.2×10^5^μm^2^ (24).

### 2.9 Prenatal biomarker identification

Receiver Operating Characteristic (ROC) curve analysis was employed to assess individual prenatal biomarkers for their predictive utility in PPPE outcome prediction. To explore the synergistic potential of 2 biomarkers together, logistic regression modeling was employed to create a combined predictive model. The ROC curve visually represents the trade-off between true positive rate (sensitivity) and false positive rate (1-specificity) across varying classification thresholds.

### 2.10 Statistical analysis

Data are presented as mean ± standard error of the mean (SEM). As appropriate, data were analyzed using the Mann-Whitney or Fisher’s exact test using GraphPad Prism, version 10.0.3 (GraphPad Software). A probability (p) value of <0.05 was considered statistically significant. A batch correction was performed for cluster comparisons, and the statistical cut-off for differentially expressed genes was p.adj<0.05, and a fold change≥ ± 1.5 and a p.adj<0.05 was used for pathway analyses.

### 2.11 Study ethical approval and funding

This work was supported by grants from Canadian Institute of Health Research - CIHR to SG and CHG, Mayo Clinic to SG, and Scholarships from the Quebec Research Network in Perinatal Determinants of Children Health and the Fonds de Recherche du Quebec – Santé to CC. Funding sources were not involved in any part of this work’s study design, analysis, interpretation, and writing. Approval was obtained from the Centre Hospitalier Universitaire (CHU) Sainte-Justine (CHUSJ) Ethics Board for patient recruitment (IRB 2015-840) in Montreal, QC, Canada, and for experiments and data analysis at the Mayo Clinic (IRB 21-012205) in Rochester, MN, USA. Patients gave written informed consent either when they were recruited either at the time of delivery with uncomplicated pregnancies or when admitted for PPPE.

## 3 Results

### 3.1 Population characteristics: demographic and obstetrical information

Ninety-six women were included in this study, all with uncomplicated pregnancies delivered at term (gestational age at delivery: 39.6 ± 0.13 CTL vs 39.1 ± 0.16 PPPE). Demographic and obstetrical characteristics are shown in **Table 1**. PPPE patients were more likely to be of black race (57.1% vs. 24.1%, p<0.01), had higher pre-pregnancy body mass index (BMI, 28.4 ± 1.0 vs. 24.9 ± 0.6 kg/m^2^, p<0.01), were more likely to have a personal and family history of hypertension and PE (33.3% vs. 1.8%, p<0.001 and 57.1% vs. 27.8%, p<0.01 respectively), and had lower gestational age at delivery (39.1 vs. 39.6 weeks, p<0.05) compared to controls. Both groups had similar maternal age, rate of primiparity, birth weight, and incidence of gestational diabetes mellitus during their pregnancy. Additionally, both groups had equal proportions of labor inductions, cesarean sections, and the proportion of male/female newborns. For women diagnosed with PPPE, the mean postpartum presentation day was 7.4 (range of 2-25 days), with the most common secondary symptoms presented in **Supplementary Figure S1**.

**Table 1:** Patient demographic and obstetrical information.

	CTL (N=54)	PPPE (N=42)
**Maternal age** (years)	34.5 (22-43)	33 (19-46)
**Race**		
White	35 (64.8)	15 (35.7)**
Black	15 (24.0)	24 (57.1)**
Others	6 (11.2)	3 (7.2)
**Pre-pregnancy BMI** (kg/m^2^)	24.9 (17.7-39.0)	28.4 (19.0-44.9)**
Overweight (>25)	21 (38.9)	27 (64.3)*
Obese (>30)	12 (22.2)	15 (35.7)
**Family history HT & PE**	15 (27.8)	24 (57.1)**
**Personal history HT & PE**	1 (1.8)	14 (33.3)***
**Primiparity**	8 (14.8)	7 (16.7)
**Birthweight** (grams)	3482 (2440-4440)	3368 (2260- 4210)
**DM current pregnancy**	9 (16.7)	10 (23.8)
**Induction of labor**	16 (29.6)	13 (30.1)
**Delivery by CS**	35 (64.8)	20 (47.6)
**Sex of the baby** (% male)	27 (50.0)	17 (40.5)
**Diagnosis** (days PP)	n/a	7.4 (2-25)

Data is presented as mean (range) or number (percentage). BMI, body mass index; HT, hypertension; PE, preeclampsia; DM, diabetes mellitus; CS, cesarean section; PP, post-partum. Statistical analyses by unpaired t-test, Mann-Whitney test, or Fisher exact test when appropriate where *p<0.05, **p<0.01, ***p<0.001.

### 3.2 Single-cell analyses identify transcriptional changes in the monocytes and dendritic cells of PPPE patients

**Figure 1:**
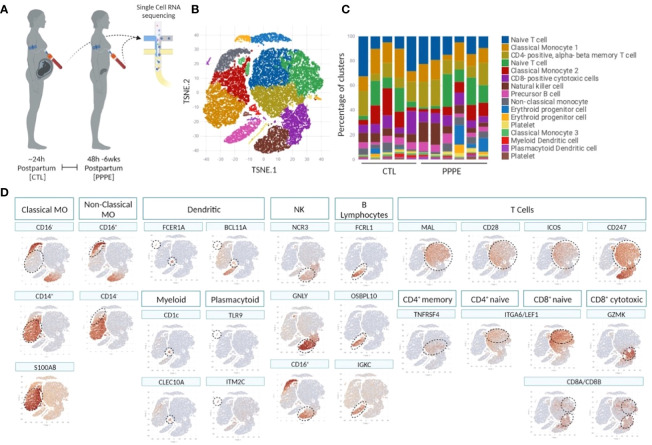
Single cell identification of immune cell populations and corresponding proportions. Selection of a subset of control (CTL; n=5) and PPPE (n=6) patients for single-cell sequencing of PBMCs **(A)** and corresponding pseudo bulk Seurat-based clustering of cells **(B)** along with the proportions of identified cells per patient **(C)**. TSNE heatmaps indicate how each main immune cell type was identified based on a signature of known markers (expression in red) **(D)**.

To deepen our understanding of the transcriptional profiles of immune cells in PPPE, we performed single-cell RNA-seq on whole PBMCs from a subset of control (n=5) and PPPE (n=6) patients (**Figure 1A**). Using pseudo-bulk Seurat graph-based clustering, our analyses uncovered 16 cell clusters, with no clustering based on fetal-sex or race (data not shown) and for which proportions were similar between patient groups and which were identified using known immune markers (**Figures 1B–D**).

#### 3.2.1 Monocytes

**Figure 2:**
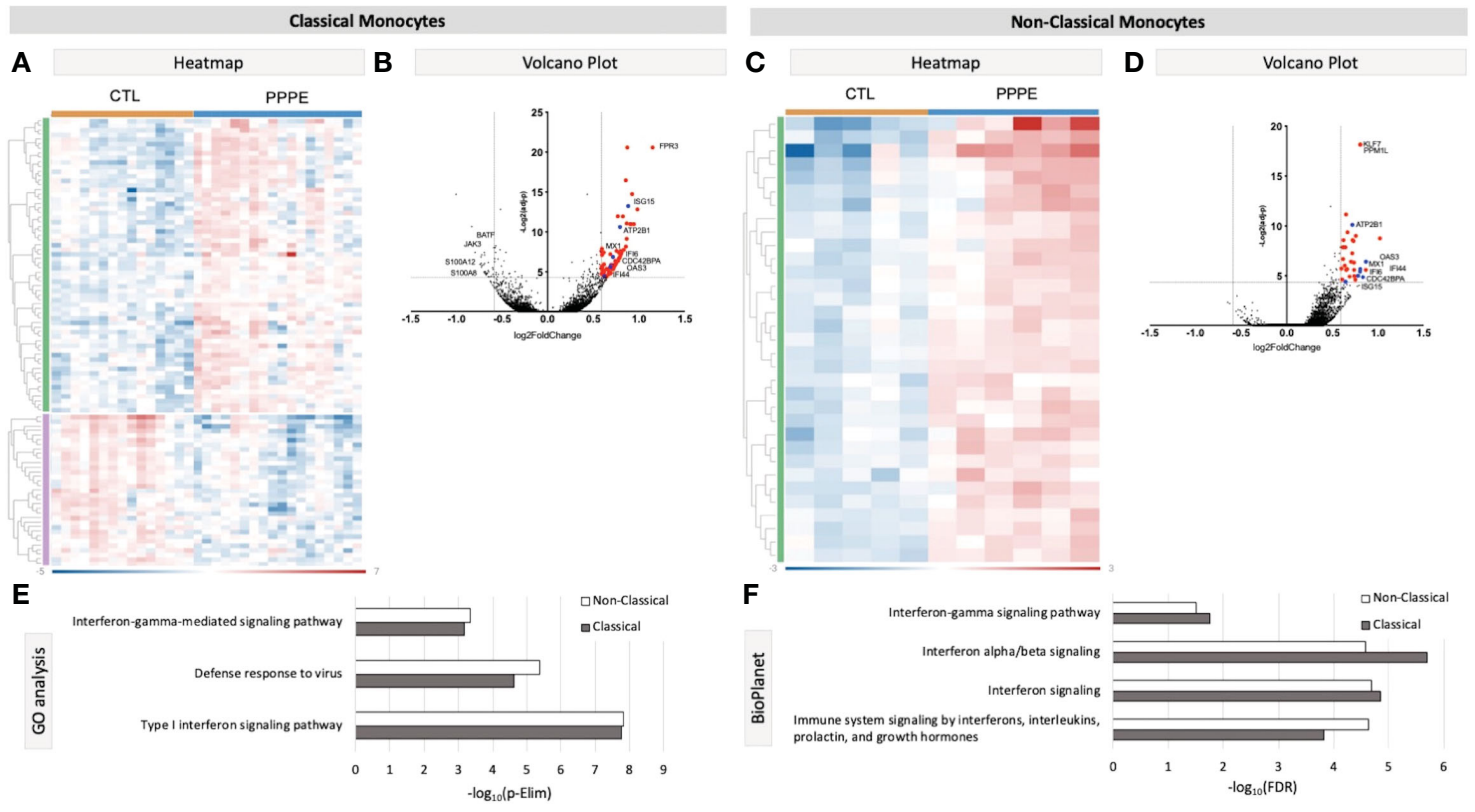
Transcriptional changes in Monocytes of PPPE patients. Classical monocytes in PPPE patients had 64 upregulated DEGs (green) and 33 downregulated DEGs (purple) represented in the heatmap **(A)** and in the volcano plot **(B)** where upregulated genes meeting cut-off criteria (red) are highlighted. Non-classical monocytes had only 33 upregulated DEGs (green) represented in the heatmap **(C)** and in the volcano plot **(D)** where upregulated genes meeting cut-off criteria (red) are highlighted. Between classical and non-classical monocytes, 7 DEGs were commonly upregulated, and these specific genes are highlighted in blue **(B, D)**. Pathway analyses revealed similar enrichment in both monocyte subtypes in PPPE using 2 complimentary catalogs: Gene Ontology analysis **(E)** and BioPlanet **(F)**. DEG cut-off is logFC±1.5 and p.ajd<0.05.

Known marker overlay on tSNE plots revealed four monocyte clusters: three classical monocyte and one non-classical monocyte clusters. Classical monocyte clusters were combined for downstream comparison analyses. Control versus PPPE cluster comparisons revealed 97 differentially expressed genes (DEGs) in classical monocytes, of which 64 were up-regulated, and 33 were downregulated in PPPE (**Figures 2A, B**). In contrast, non-classical monocytes had 33 DEGs, all up-regulated in PPPE (**Figures 2C, D**). Meta-analysis of classical and non-classical monocytes identified seven common DEGs consistently elevated in PPPE patients, namely *ATP2B1*, *CDC42BPA, ISG15, IFI44, IF16, MX1*, and *OAS3* (highlighted in blue in **Figures 2B, D**). Although the transcriptomic signatures between classical and non-classical clusters are primarily distinct, pathway analysis and gene ontology (GO) analysis both support that these PPPE monocyte subtypes have enhanced interferon signaling (**Figures 2E, F**).

**Figure 3:**
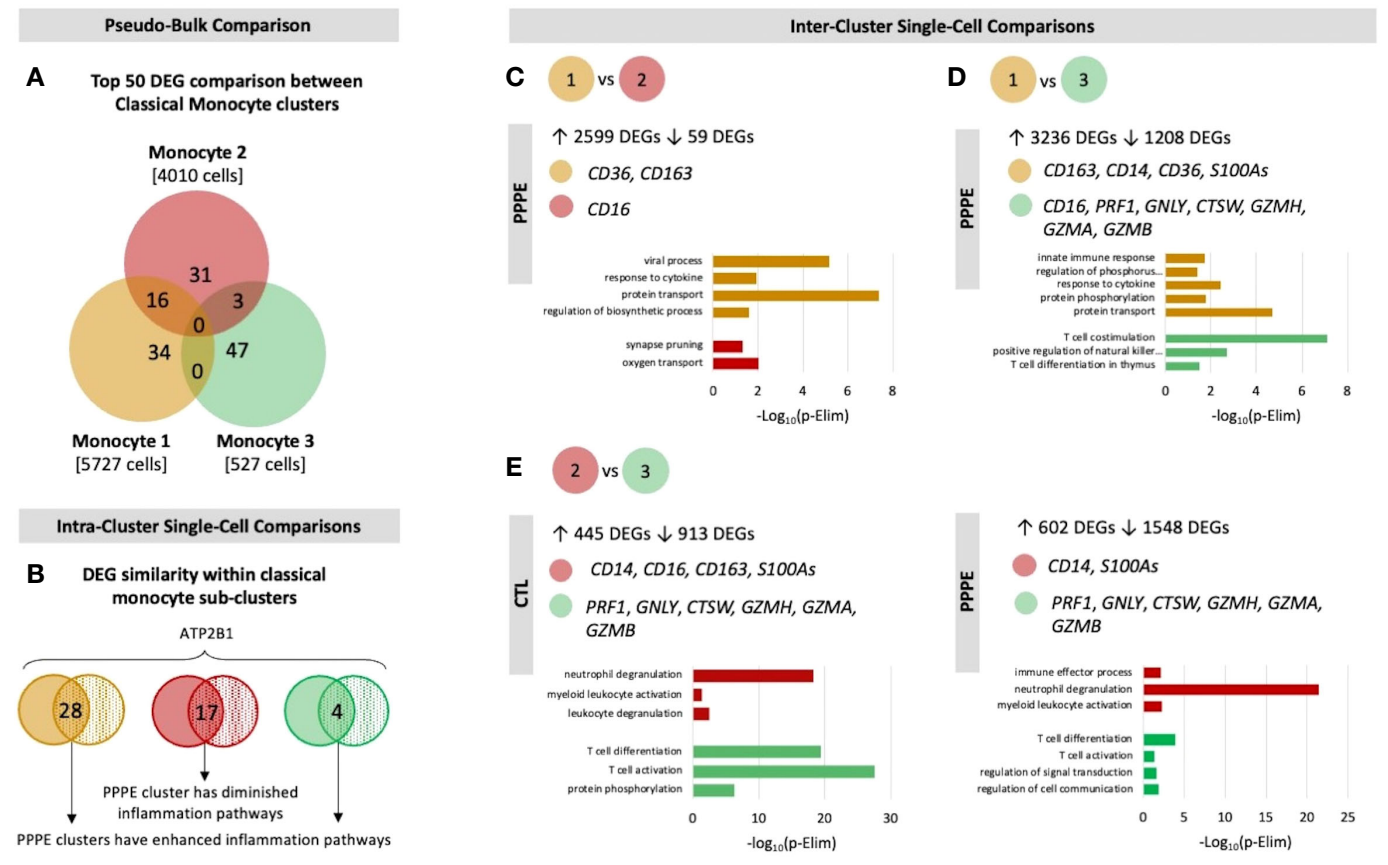
Classical monocyte sub-cluster analysis. To better understand the three classical monocyte sub-clusters a deeper analysis was performed. **(A)** At pseudo-bulk granularity, classical monocyte clusters are distinct in their top 50 most differentially expressed gene composition, where clusters 1 and 2 resemble each other the most. **(B)** At a single-cell granularity, intra-cluster analyses show that PPPE clusters 1 and 3 have enhancement of inflammation-related pathways whereas cluster 2 has no pathways enhancements. **(C-E)** With inter-cluster analyses, we find patterns of CD14 and CD16 markers between clusters 1 and 2 as well as a cytotoxic signature in cluster 3.

Previous studies have demonstrated sub-types of classical monocytes; therefore, we investigated these clusters at a higher resolution. Classical monocyte clusters 1 and 2 were the largest (5727 and 4010 cells respectively), whereas cluster 3 was small (527 cells) and most unique in terms of its DEGs (**Figure 3A**). Overall, classical monocytes from control patients were less distinct from each other, and intra-cluster comparisons point to predominant inflammation in PPPE, specifically clusters 1 and 3 (**Figure 3B**). Inter-cluster comparisons uncovered differences in gene expression, notably for CD14 and CD16, unique to PPPE. Cluster 1 showed down-regulation of CD16 expression, typical of the classical genotype (**Figures 3C, D**). On the other hand, cluster 2 had up-regulation of CD16 expression when compared to cluster 1 but not cluster 3, suggesting it could be a more intermediate-like monocyte cluster (**Figures 3C, E**). Cluster 3 was similar in control and PPPE patients whereby it differentiated itself from the other clusters with a distinct cytotoxic gene signature (*i.e., PRF1, GNLY, CTSW, GZMH, GZMA, GZMB*; **Figures 3D, E**).

#### 3.2.2 Dendritic cells

**Figure 4:**
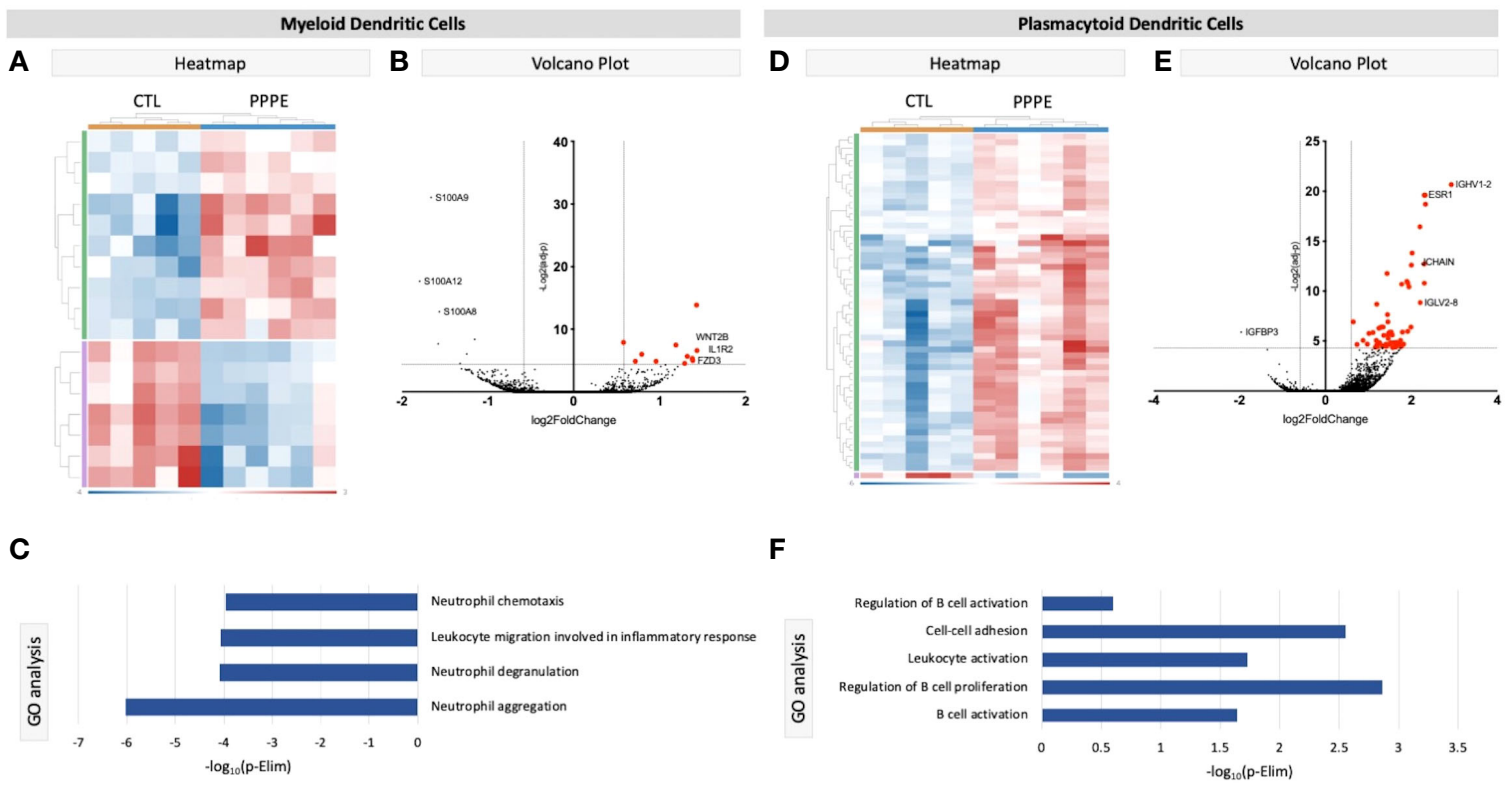
Transcriptional differences in PPPE Dendritic cell subtypes. Myeloid dendritic cells in PPPE patients had 10 upregulated DEGs (green) and 7 downregulated DEGs (purple) represented in the heatmap **(A)** and on the volcano plot **(B)**, which pointed to decreases in gene ontology biological processes relating to neutrophil activity **(C)**. Plasmacytoid dendritic cells had 57 upregulated (green) and 1 downregulated (purple) DEG represented in the heatmap **(D)** and in the volcano plot **(E)**, which resulted in B cell-related pathway enrichment **(F)**. Up-regulated DEGs meeting the cut-off criteria (logFC±1.5 and p.ajd<0.05) are highlighted in red on the volcano plots.

We identified two subtypes of dendritic cells (DC): myeloid (mDC) and plasmacytoid (pDC). Transcriptional profiles of mDC revealed 10 up-regulated DEGs in PPPE, including *IL1R2, WNT2B*, and *FZD3*, and a down-regulation of seven DEGs, including *S100A* proteins (**Figures 4A, B**). GO analyses established diminished pathways related to leukocyte migration and neutrophil activity, including chemotaxis, degranulation, and aggregation in PPPE (**Figure 4C**). On the other hand, pDCs had 57 up-regulated genes in PPPE, including estrogen receptor 1 (*ESR1*) and antigen presentation-related genes (*IGHV1-2, JCHAIN*, and *IGLV2-8*) as well as one down-regulated gene: insulin-like growth factor binding protein 3 (*IGFBP3*; **Figures 4D, E**). GO analyses demonstrate an enhancement in pathways related to the regulation of B cell activation and proliferation and cell-cell adhesion in PPPE (**Figure 4F**). All DEGs in our DC subtypes were unique and showed no overlaps.

#### 3.2.3 Other cell types

Cluster comparisons of other cell types showed minimal to no differences between our two groups. Cytotoxic CD8+ T cells revealed only one DEG, long intergenic non-protein coding RNA 1619 (*LINC01619*), similar to the comparison of naïve CD8+ T cells, which provided only one DEG: *CRIP1* (data not shown). We found no differentially expressed genes in the cluster comparisons of B cells, NK cells, or CD4+ naïve or memory T cells (data not shown).

### 3.3 Decreases in maternal circulating monocytes as part of the immune cell profile alterations in PPPE

**Figure 5:**
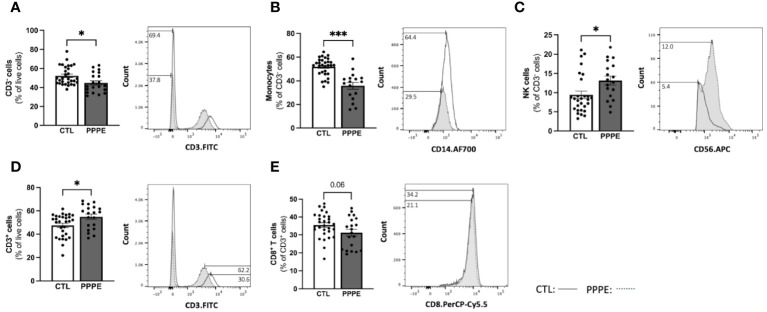
The maternal circulating immune cell profiles are altered in PPPE. Flow cytometry analysis of circulating PBMCs in CTL (n=30) and PPPE (n=20) patients showed a decrease in CD3− cells in PPPE which can be seen as a percentage of live cells in the bar graph and through the shifts of CD3 expression in representative participants in the histogram **(A)**. Monocytes were also decreased in PPPE seen as a percentage of CD3− cells in the bar graph and through the histogram of CD14 expression **(B)** along with an opposing increase in NK cells seen as a percentage of CD3- cells via bar graph and the histogram of CD56 expression **(C)**. An increase in CD3+ cells in PPPE can be seen as a percentage of live cells in the bar graph and through the shifts of CD3 expression in representative participants seen in the histogram **(D)**. CD8+ T cells were decreased in PPPE as a percentage of CD3+ lymphocytes and through the shift in CD8 expression **(E)**. Results are presented as mean ± SEM. *p<0.05, ***p<0.001.

To address changes in the maternal circulating immune system, we also analyzed the proportions of circulating PBMCs via flow cytometry from a larger subset of our patients. Here we found changes in the proportions of CD3- cells, which were decreased in PPPE (45.0 ± 2.2% vs. 52.6 ± 1.8%, p<0.05), mainly driven by the large decrease in monocytes (35.8 ± 2.8% vs. 52.2 ± 1.3%; p<0.001); whereas NK cell subsets were increased (13.1 ± 1.1% vs. 9.6 ± 0.9%, p<0.05; **Figures 5A–C**). Other CD3- subsets, such as B lymphocytes, remained unchanged (data not shown). Reciprocally, CD3+ cells were increased in PPPE (54.71 ± 2.2% vs. 47.25 ± 1.8%, p<0.05) and their CD8+ T cells subset tended to being decreased (31.21 ± 1.9% vs. 35.47 ± 1.3%; p=0.0598; **Figures 5D, E**). Other CD3+ subsets, namely NKT and CD4+ T cells (including T-regulatory and T-helper cells), remained unchanged (data not shown). No correlations were observed between the percentage of any immune cells studied with the number of days postpartum (**Supplementary Figure S2**), supporting that the observed changes are due to the pathology itself.

### 3.4 Dampened inflammatory responses in monocytes from PPPE after exposure to inflammatory stimuli

**Figure 6:**
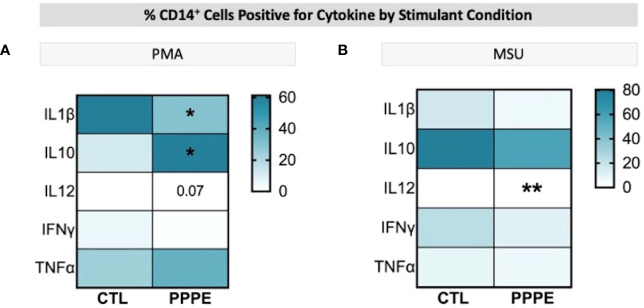
ICS of Monocytes shows a dampened inflammatory response. Heatmaps showing the percentages of CD14+ cells that are positive for staining of a specific cytokine (y-axis). When stimulated by PMA, a lower proportion of CD14+ cells were positive for IL1β and IL12, but a higher proportion were positive for IL10 **(A)**. When stimulated by MSU, these cells also showed a lowered proportion of IL12 staining **(B)**. Statistics by unpaired t-test where *p<0.05, **p<0.01.

Immune cells function primarily via the production of cytokines – we thus investigated the cytokine production profile from monocytes following exposure to classical stimuli. At baseline (unstimulated) and under LPS (pathogen-derived) stimulation, the proportion of CD14+ cells producing IL1β, IL10, IL12, and IFNγ, and the level of their production (mean fluorescence intensity - MFI), did not differ between CTL and PPPE patients (data not shown). However, under the stimulation with phorbol myristate acetate (PMA), a diminished proportion of cells from PPPE patients were found positive for pro-inflammatory cytokines IL1β and IL12 (p<0.01 and p=0.068, respectively), while an increased proportion was positive for the anti-inflammatory cytokine IL10 (p<0.01; **Figure 6A**). Similarly, exposing the cells to sterile-inflammatory stimuli, namely monosodium urate crystals (MSU), shown to be involved in PE (25–28), we found that fewer CD14+ cells produced IL12 (p<0.01, **Figure 6B**), all supportive of a dampened inflammatory response.

### 3.5 Cytokine production by T-cells supports a dampened inflammatory reaction in PPPE.

**Figure 7:**
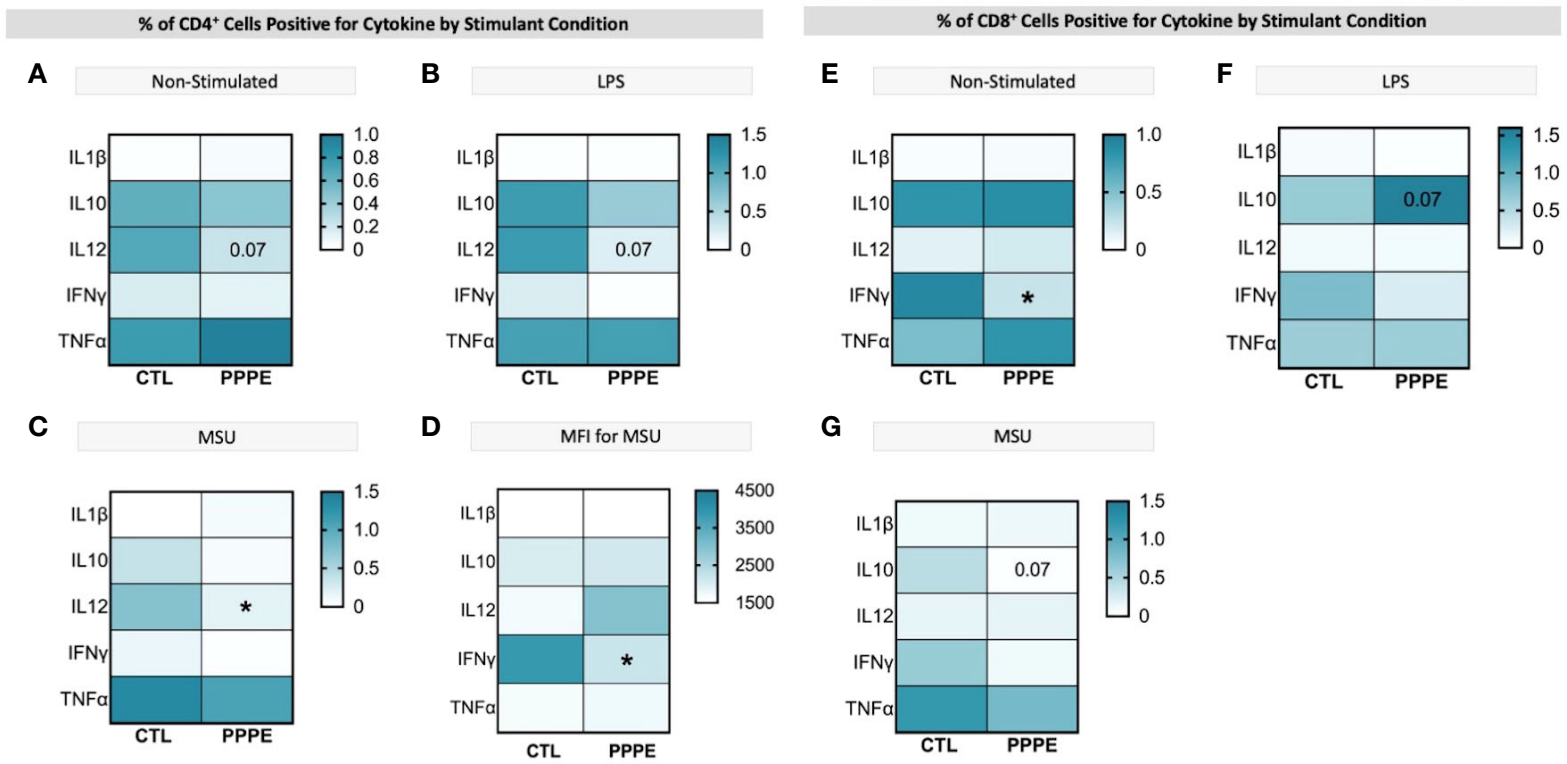
ICS of T cell subsets demonstrates a dampened inflammatory response. Heatmaps showing the percentages of CD4+ (left) and CD8+ (right) T cells that are positive for staining of a specific cytokine (y-axis). CD4^+^ T cells showed a consistently lowered proportion of cells positive for IL12 in unstimulated **(A)**, LPS-stimulated **(B)**, and MSU-stimulated **(C)** conditions. Using mean fluorescence intensity, CD4+ T cells showed overall decreased IFNG production **(D)**. CD8+ T cells under no stimulation had a lowered proportion of IFNγ positive cells **(E)** and had stimulant-dependent results for IL10 where LPS stimulation showed an increased proportion **(F)**, but MSU showed a decreased proportion of positively stained cells **(G)**. Statistics by unpaired t-test where *p<0.05.

T-cell subsets showed different cytokine production profiles in both *in vitro* LPS stimulated and unstimulated conditions in PPPE. There was a consistent trend to decreased numbers of IL12-producing CD4+ lymphocytes in PPPE patients regardless of stimulation (p=0.068, p=0.076, and p<0.05 respectively; **Figures 7A–C**). CD4+ cells also had a diminished capacity to produce IFNγ (p<0.05, **Figure 7D**) although the number of IFNγ-producing cells was unchanged. On the other hand, the number of IFNγ-producing cells was decreased in the CD8+ cell population at baseline (p<0.01; **Figure 7E**). Overall, the response in CD8+ cells was not as consistent as in CD4+ cells. Importantly, the proportions of cells producing IL10 were altered in a different direction based on the type of treatment. LPS caused a tendency to have a higher proportion of CD8+ cells producing IL10 (p=0.071; **Figure 7F**), whereas MSU led to a diminished proportion of positive cells (p=0.066; **Figure 7G**).

### 3.6 Immune cell exhaustion phenotype in PPPE

**Figure 8:**
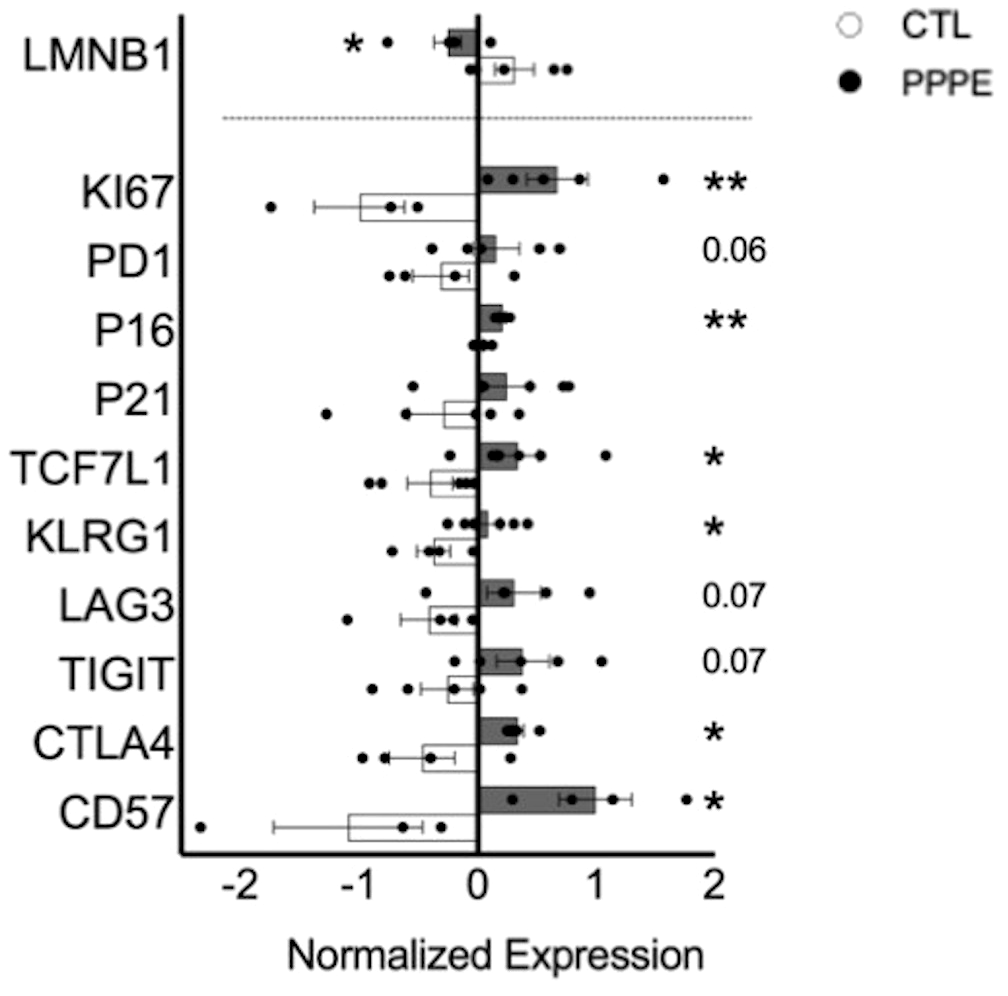
Immune cell exhaustion found in PPPE patients. PPPE patients (dark grey) demonstrated an increased immune exhaustion phenotype compared to CTL (white). This was observed via the decrease in *LaminB1* (above the dotted line in the bar graph) and through the increased expression of known exhaustion and senescence-associated markers (below the dotted line in the bar graph). Data presented as normalized expression values (averaged per condition) derived from scRNA sequencing results. Statistics by unpaired t-tests where *p<0.05, **p<0.01. Bar graph presented as mean ± SEM.

As there is a net decrease in the proportion of monocytes and T-cells producing inflammatory cytokines, and their overall levels of production, we sought to investigate if PPPE immune cells displayed a phenotype, similar to what is observed in PE, of exhaustion (29–32). Using pseudo-bulk comparisons of our sequencing data presented above, we cross-referenced specific exhaustion markers, including inhibitory receptors and senescence markers. We found that immune cells from PPPE patients have decreased *LMNB1* and increased *KI67, PD1, TCF7L1, KLRG1, LAG3, TIGIT, CTLA4*, and *CD57*, supporting an exhaustion phenotype in circulating immune cells (**Figure 8**).

### 3.7 Profiles of circulating immune mediator are altered in PPPE

**Figure 9:**
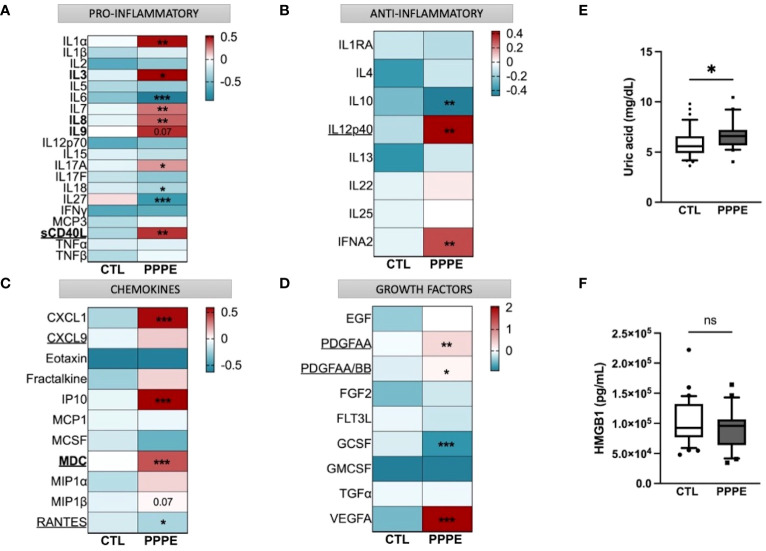
Immune mediator profiles are altered in PPPE. A multiplex panel of immune mediators in combination with ELISA analyses revealed alterations in PPPE. Circulating immune mediators were separated by category: pro-inflammatory **(A)**, anti-inflammatory **(B)**, chemokines **(C)**, or growth factors **(D)**. Data are represented by heatmaps of standardized values (standardized to CTL value) for each of the individual mediators per patient group (averaged standardized value of each analyte). We also investigated Uric acid **(E)** and HMGB1 **(F)** by ELISA and found that only Uric acid was significantly modulated. Data presented as box plots with 10-90 percentile points. Statistics done by unpaired t-test where *p<0.05, **p<0.01, ***p<0.001, ns = non-significant.

Considering the important shift in the profile of immune cells at the transcriptomic and functional (i.e., cytokine production) levels, we aimed to evaluate the profile of cytokines in the maternal circulation via a wide panel of inflammatory mediators, chemokines, and growth factors. Considering the range of time at which PPPE can be diagnosed, namely between 48 hours to 6 weeks postpartum, we first investigated if the mediators were correlated with the time of diagnosis. We found five, namely IL3, IL8, IL9, sCD40L, and MDC (presented in bold in **Figure 9**), that were correlated with the timing of diagnosis (**Supplementary Figure S3**). Seeing as hypertension is a diagnostic criterion of PPPE, we wanted to differentiate mediators that were and were not correlated to blood pressure as these could be indicative of different pathological mechanisms. We identified 7 mediators in total (underlined in **Figure 9**), of which 5 that were negatively correlated (i.e., sCD40L, CXCL9, PDGFAA, PDGFAB/BB, and RANTES), and 2 that were positively correlated (i.e., IL12p40 and MDC; **Supplementary Figure S4**). Only sCD40L and MDC were both found to be correlated to days postpartum and blood pressure.

Aside from the pro-inflammatory mediators highlighted above, three additional mediators were increased (i.e., IL1α, IL7, IL17A) and three decreased (i.e., IL6, IL18, and IL27) all independently of the timing of diagnosis, suggesting a direct association with the pathology (**Figure 9A**). We also found decreased anti-inflammatory IL10 and increased IFNα2 (**Figure 9B**). The maternal circulation in PPPE also displayed alterations in chemokines whereby there was an increase in CXCL1, IP10, and MIP1β and decreased RANTES (**Figure 9C**). Vascular endothelial growth factor (VEGFA) was increased in PPPE, but GCSF levels decreased (**Figure 9D**). We lastly analyzed uric acid and HMGB1, both of which had been associated with PE, and found that uric acid was elevated; but HMGB1 was not (**Figures 9E, F**).

### 3.8 Indications of prenatal initiation and cardiovascular implications in the PPPE pathology.

**Figure 10:**
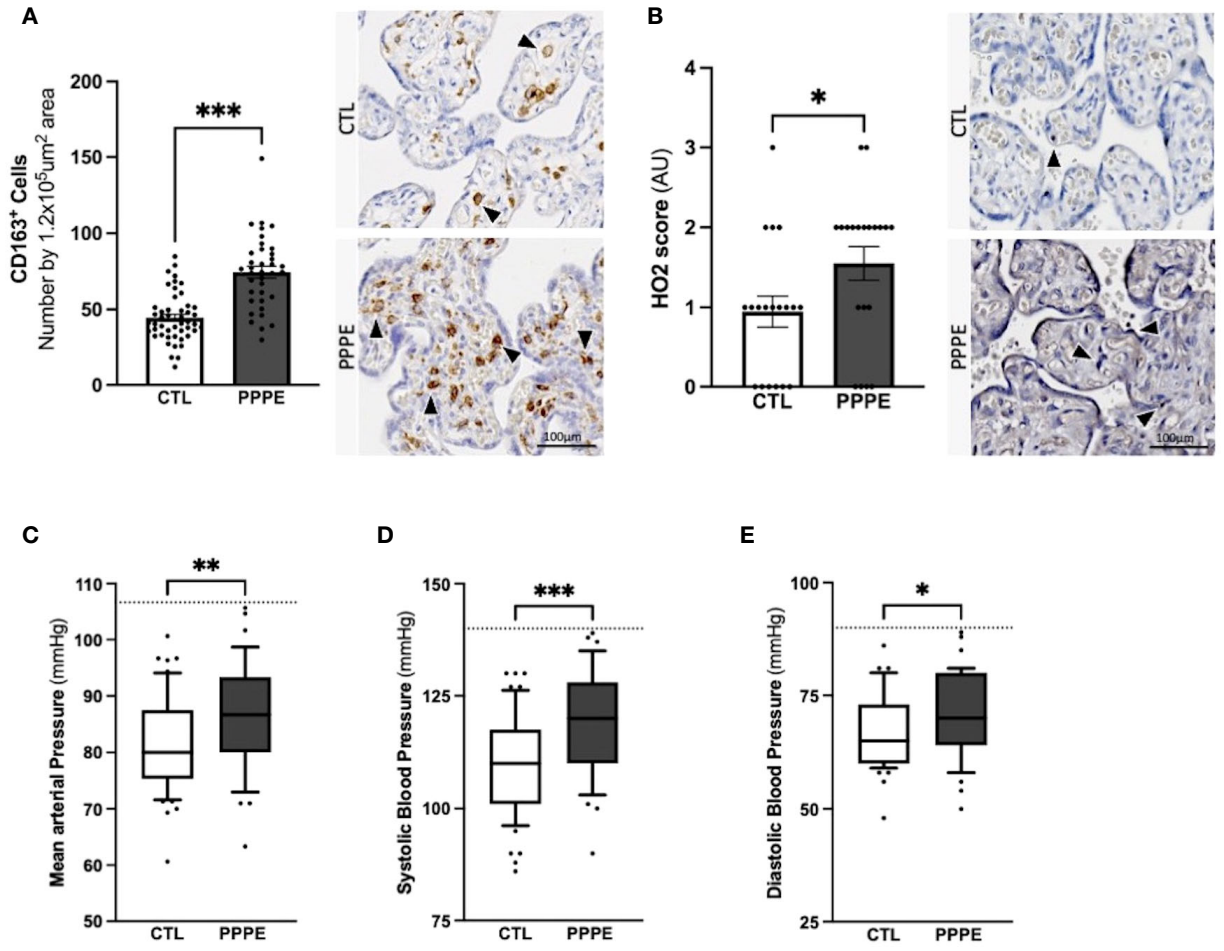
Elevated blood pressures in patients with PPPE. Immunohistological analysis of the placenta revealed that patients who went on to develop PPPE had elevated macrophage marker (CD163) **(A)** along with increased Heme Oxygenase 2 (HO2) scores **(B)**. Representative images of immunohistochemistry staining for each marker are shown for CTL and PPPE patients **(A, B)** with black and white arrowheads indicating staining. Additional signs of prenatal initiation were evidenced by blood pressures taken at the 1^st^ trimester. Mean arterial blood pressures (MAP) were calculated using the systolic and diastolic pressures and revealed that patients with clinically uncomplicated pregnancies but who later developed PPPE had higher MAP **(C)** which was independently true for both systolic **(D)** and diastolic **(E)** blood pressures, while remaining below the hypertensive cut off of 140/90mmHg (dotted line) **(C-E)**. Bar graphs are presented as mean ± SEM. Box and whisker plots are presented with 10-90 percentile points. Statistics by unpaired t-tests where *p<0.05, **p<0.01, and ***p<0.001. Scale bar: 100μm.

In addition to characterizing PPPE at the time of diagnosis, we investigated whether signs of prenatal initiation could be detected during pregnancy and used to identify women at risk for developing the pathology. We first investigated the placenta because its dysfunction is a cause of multiple pregnancy complications, and we have previously reported, in a smaller cohort, a placental increase in CD163+ cells. In our current cohort, we observed that the placentas of patients who went on to develop PPPE had skewed immune profiles with an elevated number of CD163+ cells (**Figure 10A**). We also investigated the presence of oxidative stress and autophagy markers, HO2 and LC3b respectively, and found an increase in HO2 (**Figure 10B**), but no changes in the levels of LC3b (**Suplementary Figure 6**).

We also examined patients’ blood pressure collected in the 1^st^ trimester. It was observed that although women who went on to develop PPPE did not reach the levels to be classified as hypertensive (i.e. 140/90 mmHg), they presented with significantly higher mean arterial blood pressures (MAP) in their first trimester, with independent increases in both systolic and diastolic pressures, compared to the uncomplicated pregnancies who did not develop PPPE (**Figures 10C–E**).

### 3.9 Prenatal biomarkers for the prediction of PPPE development

**Figure 11:**
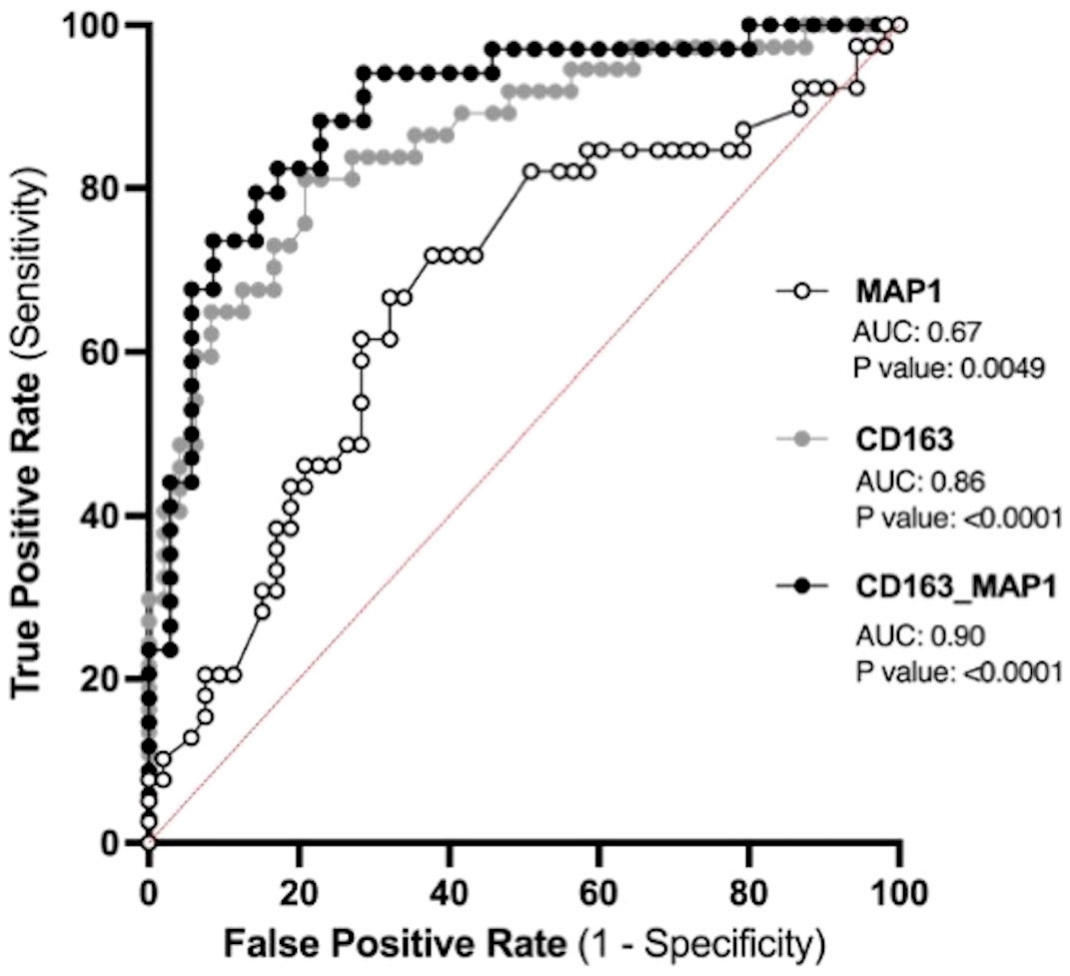
Predicting the outcome of PPPE using prenatal biomarkers via Receiver Operating Characteristic (ROC) curve analyses. ROC curves of 1^st^ trimester mean arterial blood pressure (MAP1) individually (white) and CD163 individually (grey) as well as in combination (black) following logistic regression shows strong ability to predict PPPE outcome. The ROC curve was constructed by plotting the true-positive rate (sensitivity) on the y-axis and the false-positive rate (1 specificity) on the x-axis.

The placenta is a valuable tool since samples collected before symptom onset are required for the prediction of diagnosis, and the placentas from both CTL and PPPE groups are gestational age-matched. Blood pressure measurements during the 1^st^ trimester are also valuable since they are non-invasive and consistently taken for all pregnant patients, providing insights early in gestation. Therefore, we investigated the potential use of placental CD163+ cells analysis and blood pressure in the 1^st^ trimester to identify women at risk of developing PPPE. To determine the predictive capacity of these biomarkers, we assessed them using Receiver Operating Characteristic (ROC) curves (**Figure 11**). We began with MAP at the 1^st^ trimester and found an AUC of 0.67, suggesting a reasonable but limited discriminative power. We next explored the number of CD163+ cells as another potential biomarker. We found that CD163 alone yielded a substantially higher AUC of 0.86, indicating strong predictive accuracy. A logistic regression model combining both 1^st^ trimester MAP and placental CD163, showed an improved AUC of 0.9, demonstrating excellent ability to distinguish between the CTL and PPPE, which could be used for PPPE prediction. Thresholds for individual biomarkers indicate that 83.4mmHg for the 1st trimester MAP has the strongest combination of sensitivity and specificity, both at 67%. Likewise, the threshold for CD163+ cells is 50 per defined placental area (see Methods and **Figure 10A**), which provides 84% sensitivity with 73% specificity in discriminating between patients that are CTL or going on to develop PPPE. We also investigated if MAP or numbers of CD163+ cells were driven by the differences in-between racial groups and found no differences amongst subgroups within the PPPE population (data not shown).

## 4 Discussion

Following uncomplicated pregnancies there are risks of postpartum complications such as PPPE. In the present study, we investigated the changes in the maternal circulating immune system and at the maternal-fetal interface in a sizable cohort of 42 patients who developed PPPE. We showed that these patients have altered and exhausted circulating immune profiles with a particular dysregulation in monocytes along with signs of prenatal initiation at the maternal-fetal interface. Altogether, this sets the groundwork for future preclinical and clinical research, including prospective, longitudinal studies that can now be performed in a more educated way.

In patients who developed PPPE, we detected important obstetrical and demographic differences, including race, BMI, as well as family and personal history of hypertension and PE which, in part, support previously described risk factors of the pathology (2). It has been shown that advanced maternal age and gestational diabetes mellitus are positively associated with the development of PPPE, which we did not observed, possibly due to the cohort having only late-onset PPPE in one study (2) and inclusion of patients with PE during pregnancy in the other (3). Additionally, another group has demonstrated that cesarian sections, which we did not find increased, and other habits such as physical activity, which we did not measure, are correlated to PPPE development and should be kept in mind for future work (7). We also observed changes in proportions of circulating immune cell population with PPPE having decreased CD3- and a reciprocal increase in CD3+ cells. Analysis of cell subsets found decreased monocytes and CD8^+^ T cells and increased NK cells, which partially contrasts a previous, albeit smaller study by our group, which showed an increase in monocytes, CD4+, CD8+, and NKT cell subtypes; however, we similarly found an overall increase in CD3+ and NK cells (13, 33). Looking at whole blood, it has been shown that patients with PPPE have decreased red blood cell counts, increased cholesterol and no changes in their lymphocyte or neutrophil counts compared to controls, although this was done using a haematological analyser rather than a flow cytometer (7).

Single-cell analyses supported that PPPE monocytes were also altered at the transcriptomic level. We identified 3 classical monocyte clusters that were more distinguished in PPPE, particularly cluster 1 and 2. This suggests that classical monocytes might differentiate to adapt to this altered maternal environment, which is supported by their enhancement in inflammatory pathways when compared to their control counterparts. Although not specific to this cell type, another study did identify overall inflammatory pathways when looking at the placental epigenetic dysregulation in new onset postpartum preeclampsia (1). We also identified a commonly distinct cluster 3, which had a cytotoxic gene signature resembling previously reported “natural killer dendritic cells”, in addition to co-expressing known classical monocyte genes suggesting a common role in the postpartum period (34–36).

When combining classical monocyte clusters, we found enhanced interferon signaling pathways in PPPE, which was also found in non-classical subtypes. Although monocytes are not principal producers of IFN, their production is vital in the attraction, maturation, and differentiation of leukocytes and critical in the development of type 1 T-helper cell response (37, 38). A type 1 response in PPPE was supported by alterations in circulating cytokines such as the decreased IL6, IL27, and IL10 along with increased IL12p40, IFNA2 and the IL12p70/IL10 ratio, which have been shown to favor Th1 maturation (39).

Dendritic cells were also transcriptionally dysregulated in PPPE. mDCs, which normally interact and get activated via neutrophil interactions, had diminished pathways relating to neutrophil activity, suggesting that in PPPE, these interactions are subdued and in turn, mDCs might be less activated, or exhausted (40–42). On the other hand, pDCs in PPPE patients seem more activated with enhanced pathways relating to B cell activity, which they are known to regulate (43, 44). This suggests that DCs in PPPE patients might favor an immune response with B cell action versus neutrophil action and might point to memory acquisition.

We described an altered circulating maternal environment regarding immune mediators which included shifts in pro- and anti-inflammatory cytokines, as well as chemokines and growth factors. We identified a subset of mediators that were correlated to days postpartum and/or blood pressure at the time of diagnosis which could shine light on PPPE diagnosis-related mechanisms. We also identified mediators that had no correlations and thus cannot be explained by known secondary symptoms of the pathology, for which there was a most evident increase in chemokines. Importantly, we still cannot be sure that an unidentified pathogenic mechanism might be at play and thus it remains imperative to establish normal values for these mediators in the postpartum period of control patients before shining light on their specific role in the pathology (45).

We investigated if changes in circulating mediators could be due to fluctuations in cytokine production by immune cells. We focused on the cytokine production of CD14+, CD4+, CD8+ cells and found an overall dampened inflammatory response in PPPE, with decreased pro-inflammatory and increased anti-inflammatory cytokine-production. The altered response by CD14+ cells support transcriptomic findings that monocytes react differently in the PPPE maternal environment. Unlike CD14+ cells, T cells showed baseline reduction in cytokines, such as fewer IFNγ-producing CD8+ T cells and fewer IL12-producing CD4+ T cells, which are critical to their function and survival (46). Uniquely, CD8+ T cells had opposite and stimulant-dependent IL10-production profiles, which could demonstrate a flexibility to regulate their inflammatory effects based on the context (47).

Cytokine production from immune cells is a key effector function and an impairment in this ability could be explained by exhaustion (48). Immune exhaustion in a subset of immune cells in PPPE is supported not only by diminished proportions of cytokine-producing cells, but also by the co-expression of inhibitory receptors and senescence-associated biomarkers, which were identified in our samples (48–50). It is plausible that an altered environment, although not clinically detectable, is present throughout pregnancy in patients who go on to develop PPPE, which could push cells into exhaustion.

It has been shown that T cell exhaustion is reversible; however in cases where inflammation is sustained, cell death and the development of atherosclerosis can ensue (51, 52). This supports that PPPE patients have an increased risk of cardiovascular disease later in life and is reinforced by the elevated levels of IL17A and sCD40L in the circulation of PPPE patients, both of which have been linked to myocardial infarctions, stroke, and cardiovascular deaths (53–57). These mediators have also been shown to increase the production of VEGF by endothelial cells and impact monocyte production of IL12, both of which are also found in our patients. Unlike sCD40L, VEGFA is not correlated to either days postpartum or blood pressure and has been detected at high concentrations in several cardiovascular diseases where it is associated with poor prognosis and disease severity. These findings support those of elevated serum sVCAM1 in new onset PPPE patients, indicative of endothelial dysfunction, that may lead to higher risks of cardiovascular disease later in life (7).

We additionally found that the maternal-fetal interface holds signs of prenatal changes, supporting a previous suggestion that the placenta may act as a primer in predisposed individuals (58). This included elevated heme-oxygenase and confirmed our previous finding of elevated CD163+ cells, which could be leveraged in the identification of women who develop PPPE. Heme-oxygenase works by converting heme into iron, carbon monoxide (CO), and bilirubin, and can work with CD163 to sequester hemoglobulin complexes into alternatively activated M2 macrophages (59). Localization of HO2 expression in fetal vessels (in both controls and PPPE, data not shown), suggests a possible mechanism of CO-mediated vasodilation and may protect the placenta against oxidative injury (60, 61). The increase in heme oxygenase was unexpected since it is known to be decreased in PE placentas, where its reduction is thought to play a role in placental dysfunction (62–64). This demonstrates another distinction between the pathologies and suggests that heme oxygenase could be a sign that the placenta of women who go on to develop PPPE are more stressed, and HO2 is reacting adequately to counterbalance this effect.

The prediction of PPPE is of great significance for its early prevention and treatment and for reducing maternal mortality. We aimed to find the most strategic avenue for early identification of women at high-risk of developing PPPE and thus focused on the prenatal and perinatal period. Based on our previous work (13) and our current results, this included blood pressures during gestation as well as the presence of CD163+ cells within the placenta. In contrast to our elevated blood pressure results, another study, although different in their inclusion and exclusion criteria, showed no changes in the blood pressures at admission with blood pressures elevated only at the time of discharge (3). We applied ROC to determine the predictive potential of these biomarkers individually and in combination and found that combining both in a logistic regression model could provide a highly accurate method for identifying patient at high-risk of developing the condition (AUC 0.9). Importantly, the performance of placental CD163+ cells, which is uniquely elevated in PPPE, alone showed strong potential as a predictive marker for PPPE with an AUC of 0.86 (13).

Our study includes a relatively large cohort of patients developing PPPE and contributes to the lack of knowledge pertaining to this disorder of pregnancy, that will fuel future research. Given the etiology of PPPE, a limitation of our study includes that blood samples obtained from control patients are taken close to delivery whilst those from PPPE patients are taken at the time of diagnosis, namely 48 h to 25 days after delivery. Although there are likely observable changes in the circulating immune cells in the days/weeks following delivery, within our cohort, either no differences were observed, or they were discussed in the text for correlations with days postpartum (**Supplementary Figures S2**, **S3**). In the future, prospective, longitudinal studies will be imperative to address remaining questions regarding underlying mechanisms. Lastly, although we identify biomarkers with strong predictive ability, these still need to be assessed in a validation cohort, which will be required for confirmation of prediction power.

## 5 Conclusion

Our work identifies important immune changes in the maternal circulation and at the maternal-fetal interface of patients with PPPE. Transcriptomic analyses of circulating immune cells pinpoint monocytes and dendritic cells as key players in the pathology of PPPE and immune mediator profiles support a dampened inflammatory response with immune exhaustion. We additionally found an altered placental environment that is unique in PPPE, with increased CD163+ cells that we show is a valuable biomarker for early prediction of PPPE. Understanding these changes is fundamental for guiding clinical practices for women postpartum and the ability to detect changes prenatally via the placenta and blood pressure measurements throughout gestation provides a means for identifying women who would benefit from more thorough follow-ups and risk education in the weeks following an uncomplicated pregnancy, which is not currently standard practice.

## Author’s note

Part of this work was presented at the International Federation of Placenta Associations annual meeting, Rotorua, New Zealand, September 5-8, 2023.

## Data availability statement

The datasets presented in this study can be found in online repositories. The names of the repository/repositories and accession number(s) can be found below: GSE262745 (GEO).

## Ethics statement

The studies involving humans were approved by Centre Hospitalier Universitaire (CHU) Sainte-Justine (CHUSJ) Ethics Board (IRB 2015-840), Montreal, QC, Canada; Mayo Clinic (IRB 21-012205), Rochester, MN, USA. The studies were conducted in accordance with the local legislation and institutional requirements. The participants provided their written informed consent to participate in this study.

## Author contributions

CC: Conceptualization, Data curation, Formal analysis, Investigation, Methodology, Writing – original draft, Writing – review & editing. MEB: Data curation, Writing – review & editing. JR: Data curation, Writing – review & editing. SL: Data curation, Writing – review & editing. CL-S: Data curation, Writing – review & editing. GDB: Data curation, Methodology, Writing – review & editing. IB: Data curation, Methodology, Writing – review & editing. DDS: Data curation, Methodology, Writing – review & editing. ER: Data curation, Investigation, Methodology, Writing – review & editing. SM: Data curation, Methodology, Supervision, Writing – review & editing. CG: Investigation, Methodology, Project administration, Supervision, Writing – original draft, Writing – review & editing. SG: Conceptualization, Funding acquisition, Investigation, Project administration, Resources, Supervision, Validation, Writing – original draft, Writing – review & editing, Methodology.

## Supplementary material

Supplementary Figure 1 PPPE patient symptom presentation in the postpartum period. Patients who went on to develop PPPE most often had **(A)** 2 additional symptoms at the time of diagnosis and the overall trend followed a normal distribution. From the symptoms reported, the most common was severe hypertension and headaches – the rest are listed in **(B)**.

Supplementary Figure 2 Circulating immune cells without correlations to days postpartum. Circulating immune cells that showed differences between control (CTL) and PPPE patients were not correlated to the number of days postpartum **(A**-**E)**. Data shown as simple linear regression with best-fit equation, R squared, and p values.

Supplementary Figure 3 Correlations of circulating immune mediators to days postpartum. Circulating immune mediators that showed differences between control (CTL) and PPPE patients and were also correlated to the number of days postpartum (Days PP) **(A**-**E)**. Data shown as cell type on the Y-axis and simple linear regression with best-fit equation, R squared, and p values.

Supplementary Figure 4 Correlations of circulating immune mediators to mean arterial blood pressure. A subset of immune mediators that were significantly modulated in PPPE was found to be **(A)** negatively correlated or **(B)** positively correlated to the mean arterial blood pressures. Data shown as simple linear regression with best-fit equation, R squared, and p values.

Supplementary Figure 5 Differences in prenatal markers do not show differences based on race. The number of placental CD163+ cells **(A)** as well as mean arterial blood pressures at all 3 trimesters **(B**-**D)** show no variations between are two main race populations. Data shown as either bar graphs presented as mean ± SEM or box and whisker plots presented with 10-90 percentile points. Statistics by unpaired t-tests.

Supplementary Figure 6 Signs of prenatal initiation in PPPE placenta Immunohistological analysis of the placenta revealed that patients who went on to develop PPPE had no changes in autophagy marker Microtubule Associated Protein 1 Light Chain 3 Beta (LC3b) scores. Representative images of immunohistochemistry staining for each marker are shown for CTL and PPPE patients with black and white arrowheads indicating staining. Scale bar: 100μm. Statistics by unpaired t-tests.
