# Supplementary figures and images for: Predictive biomarkers and initial analysis of maternal immune alterations in postpartum preeclampsia reveal an immune-driven pathology
Source: Front Immunol. 2024 Apr 30;15:1380629. doi: 10.3389/fimmu.2024.1380629 (PMC11091301; doi:10.3389/fimmu.2024.1380629)

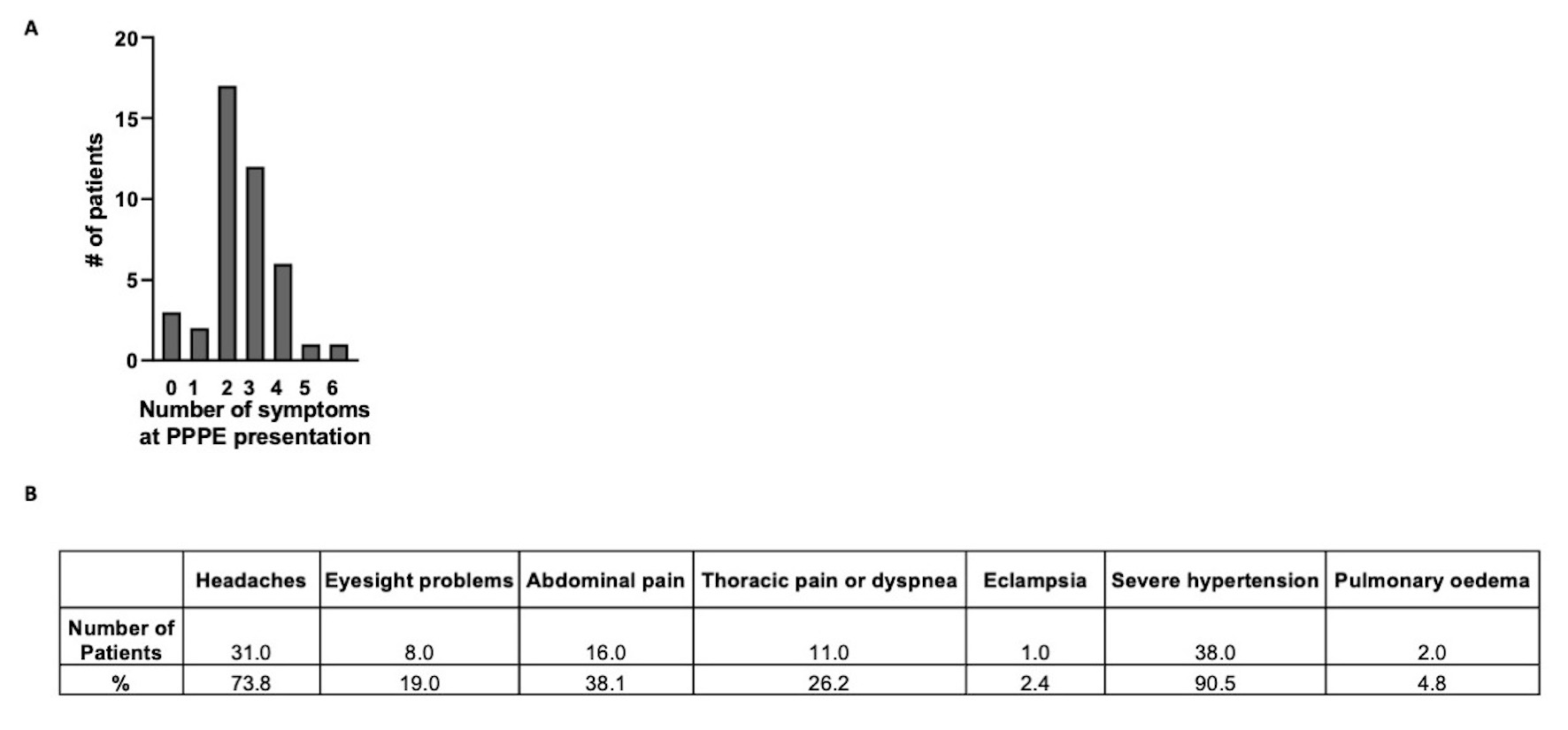

Supplement: Supplementary Figure 1 — PPPE patient symptom presentation in the postpartum period. Patients who went on to develop PPPE most often had (A) 2 additional symptoms at the time of diagnosis and the overall trend followed a normal distribution. From the symptoms reported, the most common was severe hypertension and headaches – the rest are listed in (B). [file Image_1.jpeg]

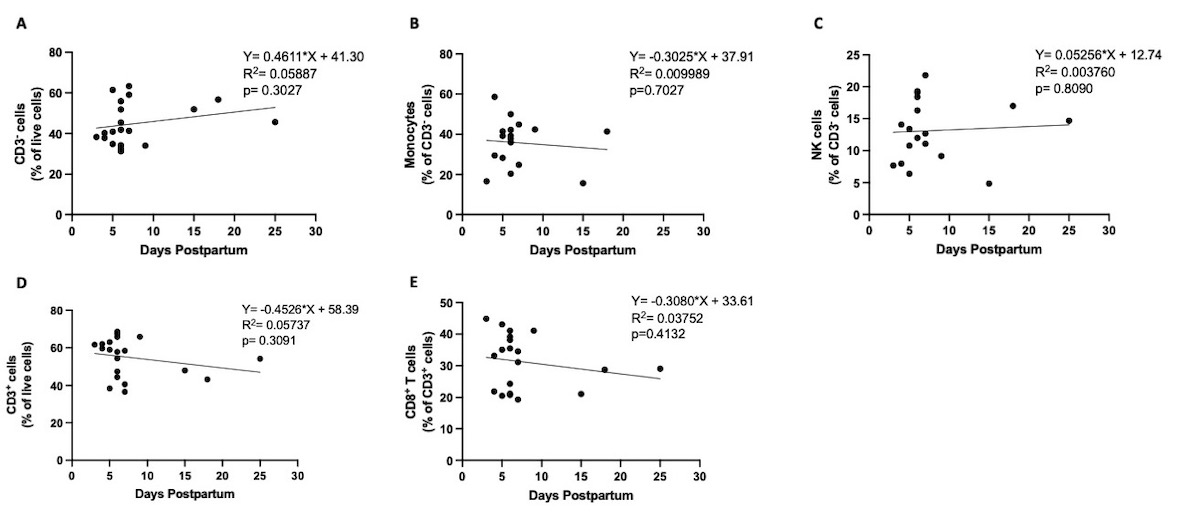

Supplement: Supplementary Figure 2 — Circulating immune cells without correlations to days postpartum. Circulating immune cells that showed differences between control (CTL) and PPPE patients were not correlated to the number of days postpartum (A-E). Data shown as simple linear regression with best-fit equation, R squared, and p values. [file Image_2.jpeg]

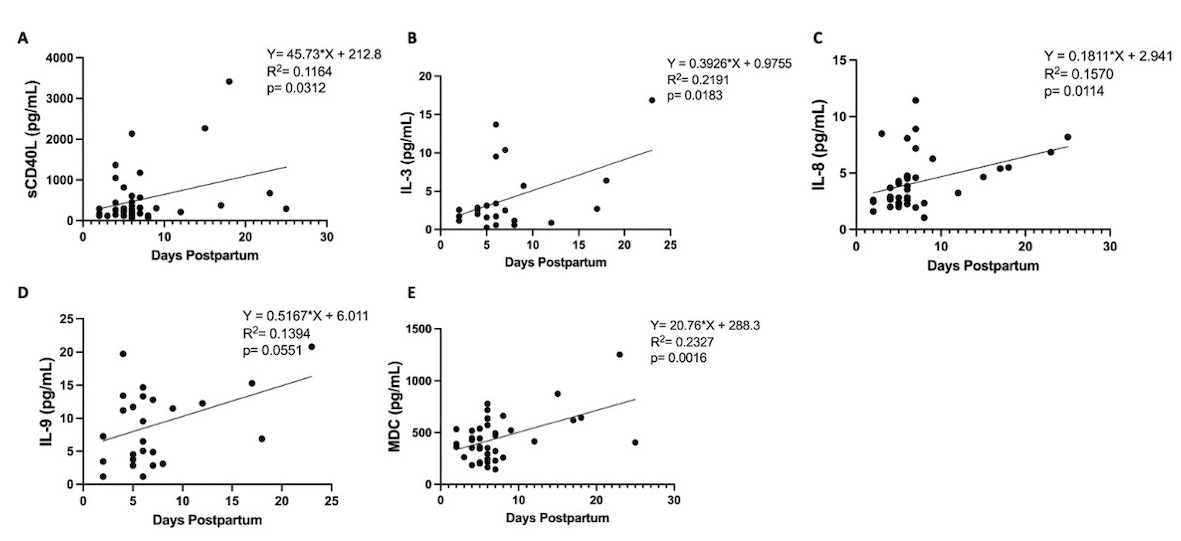

Supplement: Supplementary Figure 3 — Correlations of circulating immune mediators to days postpartum. Circulating immune mediators that showed differences between control (CTL) and PPPE patients and were also correlated to the number of days postpartum (Days PP) (A-E). Data shown as cell type on the Y-axis and simple linear regression with best-fit equation, R squared, and p values. [file Image_3.jpeg]

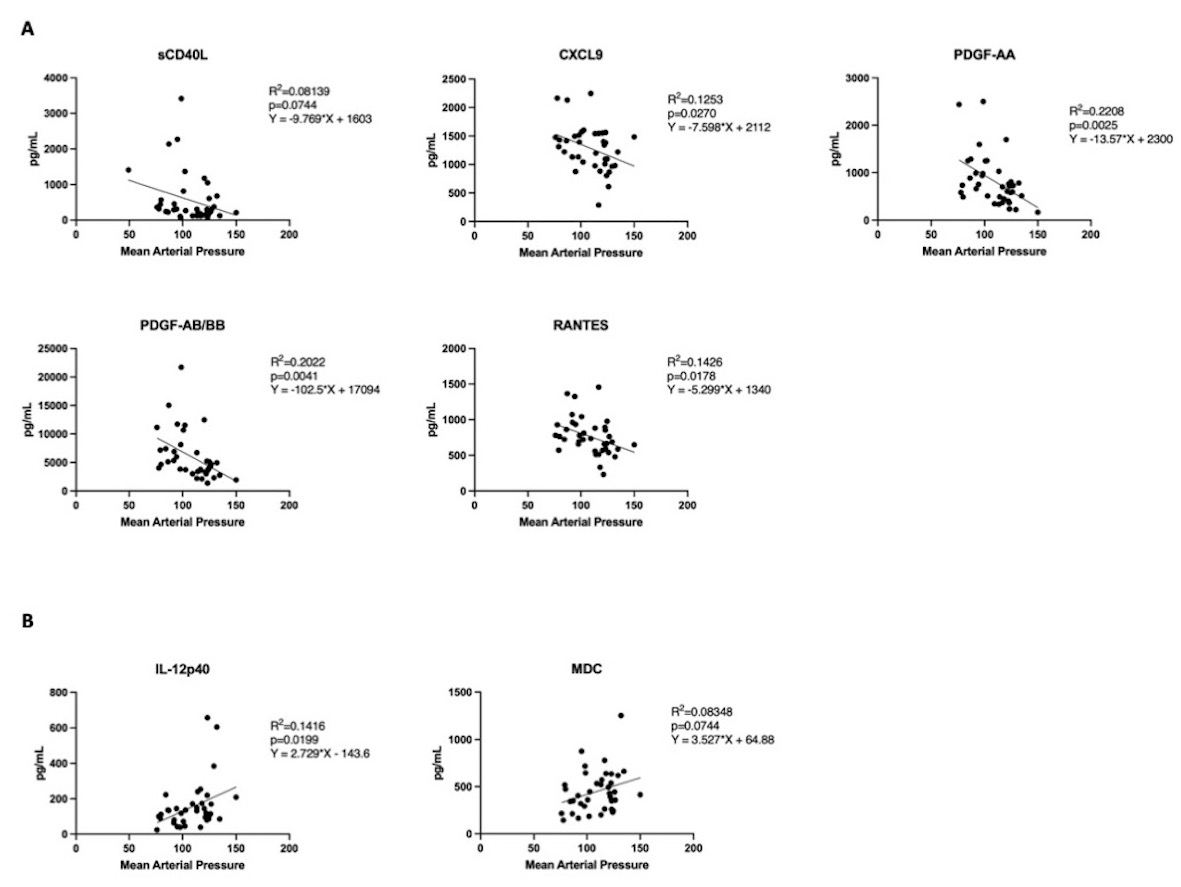

Supplement: Supplementary Figure 4 — Correlations of circulating immune mediators to mean arterial blood pressure. A subset of immune mediators that were significantly modulated in PPPE was found to be (A) negatively correlated or (B) positively correlated to the mean arterial blood pressures. Data shown as simple linear regression with best-fit equation, R squared, and p values. [file Image_4.jpeg]

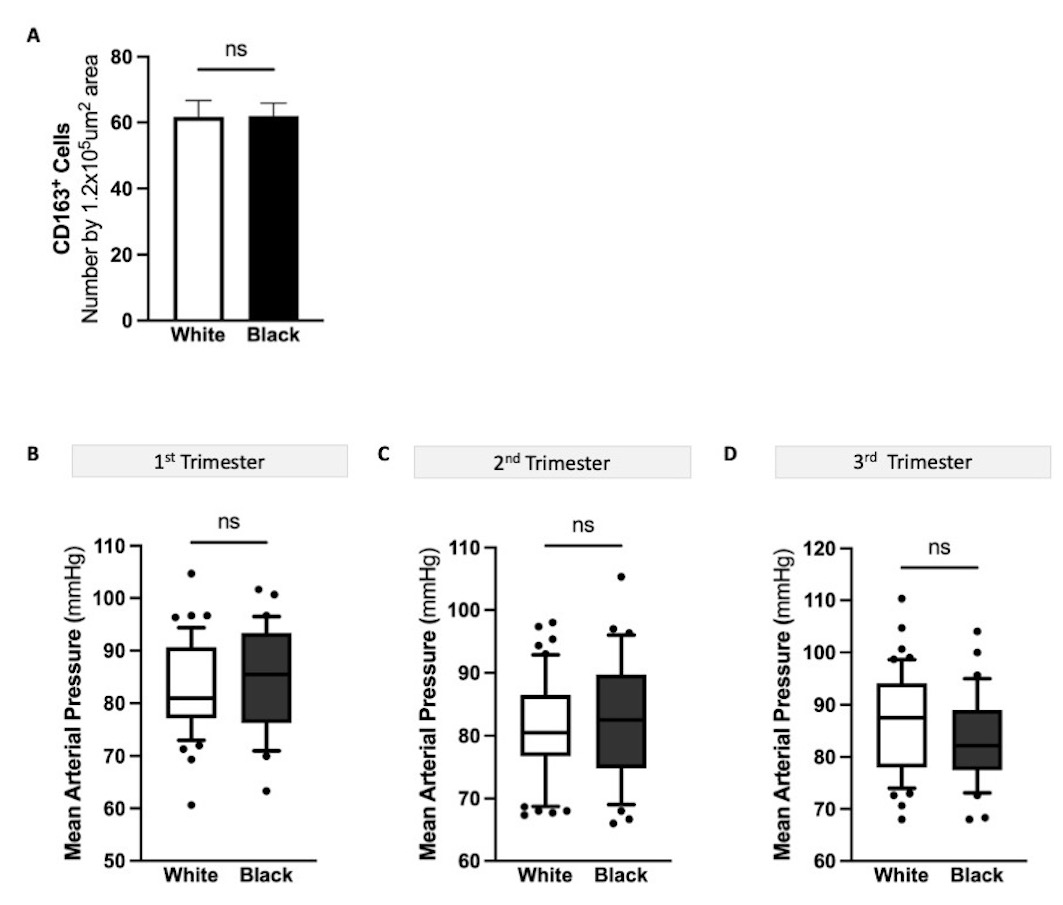

Supplement: Supplementary Figure 5 — Differences in prenatal markers do not show differences based on race. The number of placental CD163+ cells (A) as well as mean arterial blood pressures at all 3 trimesters (B-D) show no variations between are two main race populations. Data shown as either bar graphs presented as mean ± SEM or box and whisker plots presented with 10-90 percentile points. Statistics by unpaired t-tests. [file Image_5.jpeg]

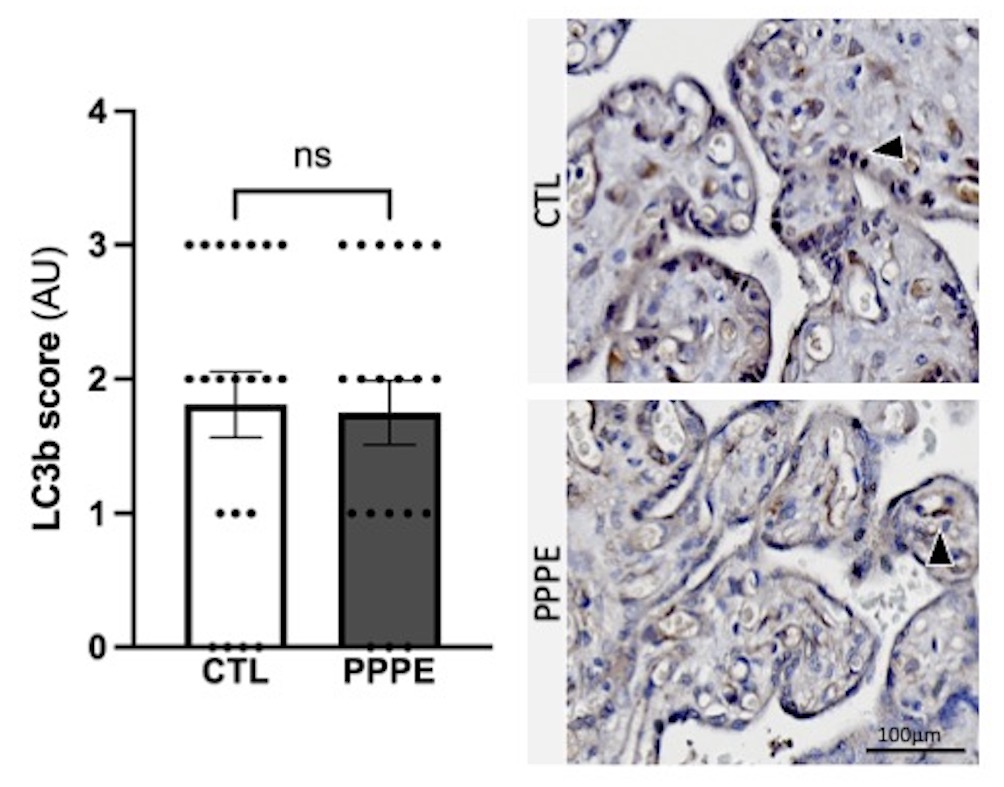

Supplement: Supplementary Figure 6 — Signs of prenatal initiation in PPPE placenta Immunohistological analysis of the placenta revealed that patients who went on to develop PPPE had no changes in autophagy marker Microtubule Associated Protein 1 Light Chain 3 Beta (LC3b) scores. Representative images of immunohistochemistry staining for each marker are shown for CTL and PPPE patients with black and white arrowheads indicating staining. Scale bar: 100μm. Statistics by unpaired t-tests. [file Image_6.jpeg]
